# Molecular epidemiology of *C. diphtheriae *strains during different phases of the diphtheria epidemic in Belarus

**DOI:** 10.1186/1471-2334-6-129

**Published:** 2006-08-15

**Authors:** Valentina Kolodkina, Leonid Titov, Tatyana Sharapa, Francine Grimont, Patrick AD Grimont, Androulla Efstratiou

**Affiliations:** 1 Belarusian Research Institute for Epidemiology and Microbiology, Minsk, Belarus; 2 Centre National de Reference pour *Corynebacterium diphtheriae*, Unite de Biodiversite des Bacteries Pathogenes Emergentes INSERM U 389, Institute Pasteur, Paris, France; 3 HPA/WHO Streptococcus and Diphtheria Reference Unit, Respiratory and Systemic Infection Laboratory, Health Protection Agency Centre for Infections, London, UK

## Abstract

**Background:**

The reemergence of epidemic diphtheria in Belarus in 1990s has provided us with important information on the biology of the disease and the diversity of the causative agent *Corynebacterium diphtheriae*. Molecular investigations were conducted with the aim to analyze the genetic variability of *C diphtheriae *during the post-epidemic period.

**Methods:**

The biotype and toxigenicity status of 3513 *C. diphtheriae *strains isolated from all areas in Belarus during a declining period of diphtheria morbidity (1996–2005) was undertaken. Of these, 384 strains were isolated from diphtheria cases, 1968 from tonsillitis patients, 426 from contacts and 735 from healthy carriers. Four hundred and thirty two selected strains were ribotyped.

**Results:**

The *C diphtheriae gravis *biotype, which was prevalent during 1996–2000, was "replaced" by the *mitis *biotype during 2001–2005. The distribution of toxigenic *C. diphtheriae *strains also decreased from 47.1% (1996) to 5.8% (2005). Changes in the distribution of the epidemic ribotypes Sankt-Peterburg and Rossija were also observed. During 2001–2005 the proportion of the Sankt-Peterburg ribotype decreased from 24.3% to 2.3%, in contrast to the Rossija ribotype, that increased from 25.1% to 49.1%. The circulation of other toxigenic ribotypes (Otchakov, Lyon, Bangladesh), which were prevalent during the period of high diphtheria incidence, also decreased. But at the same time, the proportion of non-toxigenic strains with the Cluj and Rossija ribotypes dramatically increased and accounted for 49.3% and 30.1%, respectively.

**Conclusion:**

The decrease in morbidity correlated with the dramatic decrease in the isolation of the gravis biotype and Sankt Peterburg ribotype, and the prevalence of the Rossija ribotype along with other rare ribotypes associated with non-toxigenic strains (Cluj and Rossija, in particular).

## Background

The diphtheria epidemic, which emerged in the 1990's in the newly independent states (NIS) of the former Soviet Union, was also reported from Belarus. In Belarus, 794 diphtheria cases were identified during 1990–1995, of which 25 were fatal. As a consequence of mass immunization the morbidity stabilized in 1996. In 2005 the morbidity decreased to 0.11 per 100 000 population with a morbidity index of 0.1/100 000 population in advance of the WHO target for 2010. However, the diphtheria incidence in Belarus remained higher than in previous decades. Prediction of the epidemic process and elimination of diphtheria relies mainly on pathogen circulation analysis. Microbiological monitoring currently includes modern molecular biologic and genetic approaches for the investigatation of *C. diphtheriae *isolates. A variety of molecular methods including multilocus enzyme electrophoresis, ribotyping, pulsed – field gel electrophoresis, and RAPD have been described. All these showed not only broad circulation of different genotypes but also revealed long-term persistence in regions where manifest severe forms of infections have not been registered [1,2]. The genetic population of *C. diphtheriae *was not constant throughout the epidemic process as confirmed by molecular-epidemiologic methods. The prevalence of epidemic strains that belonged to a specific biotype and ribotype was characteristic for each epidemic cycle [3,4]. Epidemic *C. diphtheriae *strains, which were prevalent during the diphtheria epidemic in Belarus, belonged to the *gravis *biotype and were represented by two ribotypes – Sankt-Peterburg and Rossija [5]. The diphtheria epidemics in Russia and the other NIS countries were also associated with these ribotypes [6-11]. Strains of the Rossija ribotype were known to have circulated in some Russian regions five years before the emergence of the epidemic [12]. The data, obtained from molecular-biologic methods, has provided much deeper understanding of the diphtheria epidemic process. However, precise mechanisms of epidemic *C. diphtheriae *strains' appearance and their elimination remain unclear. The aim of this study was to unveil changes in the *C. diphtheriae *population during the decrease of diphtheria morbidity in Belarus.

## Methods

### Bacterial strains

As a result of the implementation of microbiologic screening, a collection of *C. diphtheriae *strains circulating in Belarus was made at the Institute for Epidemiology and Microbiology in Minsk during 1996–2005. The permission for conducting the study was obtained from the Ethical Committee of the Ministry of Health of Belarus. A total of 3513 *C. diphtheriae *strains isolated from all areas in Belarus during the period of morbidity decrease were analyzed. Of these, 384 strains were isolated from diphtheria cases, 1968 – from tonsillitis patients, 426 – from contacts, and 735 – from healthy carriers.

### Biotyping and toxigenicity testing

Biotyping was performed according to the World Health Organization manual for the laboratory diagnosis of diphtheria [13]. Toxigenicity was determined by the Elek immunoprecipitation method [13]. The strains also were tested for the presence of the diphtheria toxin gene by PCR amplification (with diphtheria toxin gene-specific primers) as described in manual [13].

### Ribotyping

Total of 432 *C. diphtheriae *strains was used for ribotyping. The data for 102 strains was taken from previous study [5]. DNA was extracted by phenol/chloroform method and ribotyping of the strains was performed as previously described [5,14].

To date, 86 distinct ribotypes with the endonuclease *Bst*EII were chosen for the ribotyping database [15], and each ribotype pattern is represented by a reference strain possessing a unique geographical name, producing a stable and reproducible ribotype pattern. These reference strains also had common ribotype patterns generated by both endonucleases *Bst*EII and *Pvu*II.

Pheno- and genotyping characteristics of *C. diphtheriae *strains circulated from 1996 till 2000 correlated well with those from the 2001–2005 period.

## Results

### *C. diphtheriae *phenotypic characteristics

Analysis of 2000 *C. diphtheriae *strains, isolated in 1996–2000, showed that 60.8% belonged to the *gravis *biotype, 36.7% – to the *mitis *biotype and 2.5% – to the *belfanti *biotype (Table 1). Amongst 1513 strains isolated from 2001 to 2005, 54.4%, 39.5% and 6.1% belong to the *mitis*, *gravis *and *belfanti *biotypes respectively. Thus, the diphtheria morbidity decrease was accompanied by changes of the main *C. diphtheriae *biotypes. During, 1996–2000 the gravis biotype predominated (60.8%) and in 2001–2005 the biotype mitis predominated (54.4%).

**Table 1:** The circulation of *C. diphtheriae *biotypes in Belarus (1996–2005)

Year	Total strains	*C. diphtheriae *biotype					
		
		*gravis*		*mitis*		*belfanti*	
		
		no.	%	no.	%	no.	%
1996	486	316	65.0	168	34.6	2	0.4
1997	410	231	56.3	170	41.5	9	2.2
1998	362	209	57.7	129	35.7	24	6.6
1999	339	220	64.9	115	33.9	4	1.2
2000	403	241	59.8	152	37.7	10	2.5

**1996–2000**	**2000**	**1217**	**60.8**	**734**	**36.7**	**49**	**2.5**

2001	408	187	45.8	192	47.1	29	7.1
2002	310	130	41.9	160	51.6	20	6.5
2003	213	66	31.0	131	61.5	16	7.5
2004	290	100	34.5	169	58.3	21	7.2
2005	292	114	39.0	171	58.6	7	2.4

**2001–2005**	**1513**	**597**	**39.5**	**823**	**54.4**	**93**	**6.1**

### *C. diphtheriae *toxigenicity

Analysis of the toxigenicity of *C. diphtheriae *by the Elek test showed that during the period of decreased diphtheria morbidity there was a decline in the circulation of toxigenic *C. diphtheriae*. In 1996, toxigenic strains comprised 47.1% of 486 strains analyzed, in 2005 only 6.8% from 292 examined strains were toxigenic (P < 0.001) (Table 2). A decrease in the proportion of toxigenic strains of the two biotypes was observed: amongst *gravis *– from 65.8% to 17.5%, amongst *mitis *– from 12.5% to 0% (Table 2). In 1996–2005, 142 *belfanti *strains of a total of 3513 were non toxigenic. However, isolates of toxigenic *gravis *biotype were prevalent among toxigenic strains during the whole period. The proportion ranged from 71.4% – 100.0%.

**Table 2:** The circulation of toxigenic *C. diphtheriae *in Belarus (1996–2005)

Year	*C. diphtheriae*									Total strains		
				
	*gravis*			*mitis*			*belfanti*					
	
	total strains	tox ^+^		total strains	tox ^+^		total strains	tox ^+^		no.	tox ^+^	
								
		no.	%		no.	%		no.	%		no.	%
1996	316	208	65.8	168	21	12.5	2	0	0	486	229	47.1
1997	231	137	59.3	170	12	7.0	9	0	0	410	149	36.3
1998	209	71	34.0	129	0	0	24	0	0	362	71	19.6
1999	220	99	45.0	115	5	4.3	4	0	0	339	104	30.7
2000	241	115	47.7	152	5	3.3	10	0	0	403	120	29.8

**1996–2000**	**1217**	**630**	**51.8**	**734**	**43**	**5.9**	**49**	**0**	**0**	**2000**	**673**	**33.7**

2001	187	54	28.9	192	1	0.5	29	0	0	408	55	13.5
2002	130	25	19.2	160	0	0	20	0	0	310	25	8.1
2003	66	12	18.2	131	4	3.1	16	0	0	213	16	7.5
2004	100	40	40.0	169	16	9.5	21	0	0	290	56	19.3
2005	114	20	17.5	171	0	0	7	0	0	292	20	6.8

**2001–2005**	**597**	**148**	**24.8**	**823**	**21**	**2.6**	**93**	**0**	**0**	**1513**	**169**	**11.2**

### *C. diphtheriae *strains genetic characteristics

Genotypic characteristics of the *C. diphtheriae *population was based upon ribotype analysis of 432 strains, including 269 toxigenic and 163 non-toxigenic strains. Amongst these, 220 were from diphtheria cases, 116 – from tonsillitis patients, 45 – from contacts, 51 – from healthy carriers. Twenty ribotypes were identified amongst 259 strains, isolated during 1996–2000 (Table 3). Approximately 49.4% were attributed to the two ribotypes: Sankt-Peterburg (24.3%) and Rossija (25.1%). The remainder (50.6%) were represented by 18 ribotypes, which occurred with a frequency range of 0.4 to 16.9. During 2001–2005, the numbers of circulating *C. diphtheriae *ribotypes decreased to 12, with the Rossija (49.1%) and Cluj ribotypes (20.8%) being prevalent. Ten ribotypes represented the remaining 30.1% strains, which occurred within a frequency range of 0.6 to 11.0%. Evidence from our investigations showed that eight ribotypes, which were prevalent in earlier years, were not identified in the country during 2001–2005. At the same time the circulation level of the Sankt-Peterburg ribotype, which was the predominant epidemic genotype, decreased from 24.3% to 2.3%. This resulted in a comparative increase of the Rossija epidemic ribotype from 25.1% to 49.1%, (P < 0,001) amongst the *C. diphtheriae *population.

**Table 3:** *C. diphtheriae *ribotype prevalence in Belarus (1996–2005)

International designation	Former designation (London)	1996–2000 period		2001–2005 period	
		
		Total strains	%	Total strains	%
Sankt-Peterburg	D1	63	24.3	4	2.3
Rossija	D4	65	25.1	85	49.1
Rossija derivation		-	-	8	4.6
Otchakov	D7	18	6.9	19	11.0
Lyon	D6	11	4.2	3	1.7
Bangladesh	D36	2	0.8	1	0.6
Erlabrunn	D15	2	0.8	-	-
Schwarzenberg	D13	2	0.8	1	0.6
Moskva		44	16.9	8	4.6
Cluj	D10	34	13.1	36	20.8
Minsk	D30	1	0.4	-	-
Gomel		1	0.4	-	-
Ras-el-Ma		2	0.8	-	-
Thailand	D19	1	0.4	-	-
Prahova	D17	1	0.4	-	-
Dagestan	D9	1	0.4	-	-
Gatchina	D5	1	0.4	-	-
Buzau		1	0.4	-	-
Close to Nan	D63	2	0.8	-	-
Close to Pakistan	D12/D31	-	-	2	1.2
Close to Versailles	D44/54/55	1	0.4	-	-
Neamt	D22	-	-	1	0.6
New 1–11		6	2.3	5	2.9

Total		259	100.0	173	100.0

Analysis of the toxigenicity characteristics amongst the various *C. diphtheriae *ribotypes demonstrated that despite a dramatic decrease in the circulation of toxigenic strains during the period of declining morbidity, the *C. diphtheriae *ribotypes that predominated were still toxigenic. During 2001–2005, all strains belonging to the Sankt-Peterburg (4 strains), Otchakov (19 strains), Lyon (3 strain), Bangladesh (1 strain) exhibited toxigenic activity (Table 4). A decrease in the proportion of toxigenic strains belonging to the Rossija ribotype (from 93.8% to 74.1%) as well as that of a new ribotype, which was not prevalent in previous years, was reported. We therefore, conclude that in 2001–2005 not only "toxigenic" ribotypes which rarely occurred in the epidemic years were eliminated, but also the proportion of "toxigenic" ribotypes that were prevalent during the high incidence peak decreased from 55.6% to 27.0%. However, the strains of these ribotypes that continued to circulate remained toxigenic.

**Table 4:** Toxigenic strains amongst *C. diphtheriae *ribotypes (Belarus, 1996–2005)

Ribotype/*Bst*EII		1996–2000 period			2001–2005 period		
International designation	Former designation (London)	Total strains	Toxigenic strains		Total strains	Toxigenic strains	
			
			no.	%		no.	%

Sankt-Peterburg	D1	63	62	98.4	4	4	100.0
Rossija	D4	65	61	93.8	85	63	74.1
Rossija derivation		-	-	-	8	8	100.0
Otchakov	D7	18	18	100.0	19	19	100.0
Lyon	D6	11	11	100.0	3	3	100.0
Bangladesh	D36	2	2	100.0	1	1	100.0
Erlabrunn	D15	2	1	50.0	-	-	-
Schwarzenberg	D13	2	2	100.0	1	-	-
Moskva		44	-	-	8	-	-
Cluj	D10	34	1	2.9	36	-	-
Minsk	D30	1	1	100.0	-	-	-
Gomel		1	1	100.0	-	-	-
Ras-el-Ma		2	-	-	-	-	-
Thailand	D19	1	-	-	-	-	-
Prahova	D17	1	-	-	-	-	-
Dagestan	D9	1	-	-	-	-	-
Gatchina	D5	1	-	-	-	-	-
Buzau		1	1	100.0	-	-	-
Close to Nan	D63	2	2	100.0	-	-	-
Close to Pakistan	D12/D31	-	-	-	2	-	-
Close to Versailles	D44/54/55	1	-	-	-	-	-
Neamt	D22	-	-	-	1	-	-
New 1–11		6	6	100.0	5	2	40.0

Total		259	169	65.3	173	100	57.8

Distinct features amongst various *C. diphtheriae *ribotypes were also observed amongst non-toxigenic strains. In 1996–2000 the proportion of non-toxigenic strains from 259 analyzed, accounted for 34.7%, and increased to 42.2% amongst 173 strains examined during 2001–2005. Non-toxigenic *C. diphtheriae *strains isolated in 1996–2000 were attributed to 11 ribotypes (Table 5). The Moskva (48.9%) and Cluj (36.7%) ribotypes were prevalent within this group. The remainder of non-toxigenic strains (14.4%) was represented by nine ribotypes and occurred at a frequency of 4.5 % – 1.1%. In 2001–2005 the numbers of circulating non-toxigenic *C. diphtheriae *ribotypes decreased to seven with the Cluj (49.3%) and Rossija (30.1%) ribotypes predominating. Thus, in 2001–2005, the rare ribotypes (Ras-el-Ma, Thailand, Prahova, Dagestan, and Gatchina) were eliminated and other more rarely encountered ribotypes (Close to Pakistan, Neamt) emerged. Strains of the Cluj ribotype continued to prevail within the *C. diphtheriae *population whilst the proportion of the Moskva ribotype decreased to 11.0%. A relative increase of non-toxigenic strains was observed amongst the Rossija ribotype and as a result were second to the Cluj ribotype.

**Table 5:** Ribotypes of non-toxigenic *C. diphtheriae *(Belarus, 1996–2005)

Ribotype/*Bst*E II		1996–2000 period		2001–2005 period	
International designation	Former designation (London)	total strains	%	total strains	%

Sankt-Peterburg	D1	1	1.1	-	-
Rossija	D4	4	4.5	22	30.1
Moskva		44	48.9	8	11.0
Cluj	D10	33	36.7	36	49.3
Erlabrunn	D15	1	1.1	-	-
Schwarzenberg	D13	-	-	1	1.4
Ras-el-Ma		2	2.2	-	-
Thailand	D19	1	1.1	-	-
Prahova	D17	1	1.1	-	-
Dagestan	D9	1	1.1	-	-
Gatchina	D5	1	1.1	-	-
Close to Pakistan	D12/D31	-	-	2	2.7
Close to Versailles	D44/54/55	1	1.1	-	-
Neamt	D22	-	-	1	1.4
New 9–11		-	-	3	4.1

Total		90	100.0	73	100.0

## Discussion

The morbidity of diphtheria in Belarus decreased as a consequence of mass immunization and was accompanied in 1996 by changes in the circulating population of *C. diphtheriae*. The *gravis *biotype which prevailed in 1996–2000 was 'replaced' with the *mitis *biotype in 2001–2005. This phenomenon of biotype replacement was may be due to colonization resistance by the human population to a single biotype whilst remaining susceptible to the others [16].

Simultaneously, we observed a decrease in the proportion of toxigenic *C. diphtheriae *strains from 47.1% (1996) to 6.8% (2005). Toxigenic *C. diphtheriae *strains offer some selective advantages as compared to non-toxigenic variants in the non-immune human population. The diphtheria toxin induces local tissue changes promoting the colonization and maximum reproduction of bacteria from a clonal group thus contributing to better transmission. These toxigenic strains advantages are not found in immunized individuals [1]. This appears to be a possible explanation for the decrease in the circulation of toxigenic *C. diphtheriae *strains in a highly immune population.

Ribotyping analysis revealed the elimination of rare ribotypes (toxigenic as well as non-toxigenic) during the period of decreased morbidity. Thus, several ribotypes present 0.8–0.4% during 1996–2000 were completely eliminated. These include Minsk, Gomel, Ras-el-Ma, Thailand, Prahova, Dagestan, Gatchina, Close to Nan, Close to Versailles ribotypes. As regards *gravis *biotype strains, in 2001–2005 only the Sankt-Peterburg ribotype population dramatically decreased from 24.3% to 2.3%, in contrast, the proportion of the Rossija biotype increased from 25.1% to 49.1%. It is generally believed that surface structures of *C. diphtheriae *– which are putative colonization factors – display intraspecies differences [16]. Presumably, this could explain the complete disappearance of Sankt-Peterburg ribotype strains whilst preserving another epidemic ribotype – Rossjia. There was a significant increase in the proportion of non-toxigenic strains amongst the total circulating *C. diphtheriae *with the prevalent ribotypes being Cluj and Rossija (49.1% and 20.8%, respectively) and correlated with the long-term circulation of non-toxigenic strains of the ribotypes Rossija and Cluj. On the other hand, the Rossija ribotype demonstrated a high level of toxigenic strains during 2000–2005 (36.4% of total analysed, 63.0% of total toxigenic strains). Interestingly, a high level of toxigenic strains (100%) was also registered for the Otchakov, Lyon and Bangladesh ribotypes, although their occurrence during 2001–2005 decreased markedly.

In recent years in Belarus, population immunity has increased (92.4% of protected individuals), but the continued circulation of toxigenic *C. diphtheriae *does not exclude the emergence of sporadic cases of disease, in certain risk groups. A WHO meeting in 1993 concluded that to achieve the elimination of diphtheria, a minimum immunization coverage rate of 90% in children and 75% in adult is required. [17]. From the data available to date it is still unclear whether highly virulent and toxigenic strains will be eliminated from *C. diphtheriae *population. Rappuoli et al. [18] suggested that epidemic strains had some selective advantage, such as increased virulence or enhanced ability to colonize and spread. Unfortunately, microbial factors that distinguish epidemic from endemic strains have not been identified [19,20]. Intense investigation of advantage-giving virulence factors is necessary for epidemic strains – this will allow us to identify conditions necessary for their elimination. Further monitoring of *C. diphtheriae *circulation in Belarus with molecular-genetic methods as well as determination of molecular-genetic properties in the pathogen population will be the focus of future investigations.

## Conclusion

Diphtheria morbidity decreased in Belarus, which was accompanied by significant population changes in the genetic structure of *C. diphtheriae*. Certain correlations between the genetic evolution of *C. diphtheriae *and toxin-production have been established.

## Competing interests

The authors declare that they have no competing interests.

## Authors' contributions

PADG and FG did the riboryping of *C. diphtheriae*. AE managed the project. TS collected and provided bacterial isolates. LT wrote the report. VK did the biotyping, toxigenicity of *C. diphtheriae*, data analysis, wrote the report. All authors read and approved the final manuscript.

## Pre-publication history

The pre-publication history for this paper can be accessed here:


